# A correlation of serum fibroblast growth factor 21 level with inflammatory markers and indicators of nutritional status in patients with inflammatory bowel disease

**DOI:** 10.3389/fphys.2024.1394030

**Published:** 2024-06-25

**Authors:** Agata Łukawska, Agata Mulak

**Affiliations:** Department of Gastroenterology and Hepatology, Wroclaw Medical University, Wroclaw, Poland

**Keywords:** inflammatory bowel disease, fibroblast growth factors, disease activity, fecal calprotectin, malnutrition

## Abstract

**Background:**

Fibroblast growth factor 21 (FGF21) is a stress-inducible hormone that regulates nutrient and metabolic homeostasis. Inflammatory state is one of the stimulators of FGF21 secretion. The aim of the study was to assess correlations between serum FGF21 level and inflammatory markers as well as nutritional status indicators in patients with inflammatory bowel disease (IBD).

**Methods:**

Fasting serum FGF21 level was measured using ELISA test in 105 IBD patients and 17 healthy controls. There were 31 subjects with active ulcerative colitis (UC), 16 with inactive UC, 36 with active Crohn’s disease (CD), and 22 with inactive CD. Clinical and endoscopic activity of IBD was evaluated based on validated scales and indices. Fecal calprotectin, serum CRP, and selected parameters of nutritional status were tested in all patients.

**Results:**

Serum FGF21 level was characterized by fluctuations depending on the IBD activity. FGF21 level was significantly higher in both active UC and CD compared to inactive phases of the diseases and to the controls. A correlation between FGF21 and fecal calprotectin levels was also found in UC and CD. Additionally, in CD, FGF21 level positively correlated with CRP level. In both UC and CD, a negative correlation was noted between FGF21 level and nutritional status parameters including cholesterol, protein, albumin levels, and BMI.

**Conclusion:**

The intensity of intestinal inflammation is related to FGF21 level, which correlates negatively with nutritional status indicators in IBD. The disturbances in FGF21 secretion may contribute to the multifactorial pathogenesis of malnutrition and weight loss in IBD patients.

## 1 Introduction

The family of fibroblast growth factors (FGF) consists of 22 structurally related peptides with a diverse range of cellular functions (Beenken and Mohammadi, 2009; Degirolamo et al., 2016). Among them, there is a group of three factors with endocrine properties – FGF19, FGF21, and FGF23 (Łukawska and Mulak, 2022). These endocrine FGFs are released into the bloodstream and exert their effects on distant tissues regulating multiple metabolic processes (Beenken and Mohammadi, 2009; Degirolamo et al., 2016; Łukawska and Mulak, 2022). The function of endocrine FGFs depends on the presence of their receptors and co-receptors α-Klotho or β-Klotho. The co-receptors expression in target organs determines the tissue-specific action of the FGFs (Degirolamo et al., 2016).

FGF21 is a protein produced mainly in the liver, adipose tissue, muscles, and the pancreas. FGF21 requires the presence of β-Klotho to activate appropriate receptors in target tissues. FGF21 is involved in the metabolism of lipids, carbohydrates, and proteins. It also participates in energy expenditure and body weight regulation (Dolegowska et al., 2019). The main inducers of the FGF21 expression include fasting state, overfeeding, inflammation, and physical activity (Martínez-Garza et al., 2019). The mode of FGF21 action is considered not only endocrine but also paracrine and autocrine (Martínez-Garza et al., 2019). One of the main target organs of FGF21 is white adipose tissue (WAT). FGF21 can both inhibit and stimulate lipolysis in WAT, after meals and during fasting, respectively (Xie et al., 2020). Additionally, during fasting, FGF21 stimulates gluconeogenesis, ketogenesis, and fatty acid oxidation (Gadaleta et al., 2011). Interestingly, FGF21 may cross the blood-brain barrier and exert effect at the central nervous system related to glucose homeostasis and body weight regulation (Lan et al., 2017). Moreover, FGF21 induces the production of corticotropin-releasing hormone activating the hypothalamic-pituitary-adrenal axis and increasing gluconeogenesis in the liver during prolonged starvation (BonDurant and Potthoff, 2018). The results of previous experimental and clinical studies have shown that inflammatory stimuli may also induce the FGF21 expression (Feingold et al., 2012; Gariani et al., 2013).

Inflammatory bowel disease (IBD) is a chronic recurrent immune-mediated disorder of the gastrointestinal tract that encompass ulcerative colitis (UC) and Crohn’s disease (CD) (Zhang and Li, 2014). IBD is characterized by a wide spectrum of intestinal and extra-intestinal symptoms as well as systemic complications. The disease progresses with periods of flares and remissions (Torres et al., 2017; Ungaro et al., 2017). Due to intestinal inflammation, diarrhea, malabsorption, dietary limitations, and anorexia, patients with IBD are at increased risk of malnutrition, the prevalence of which among that group of patients ranges from 20% to 85% (Balestrieri et al., 2020).

The results of previous studies in animal models have suggested that FGF21 as a metabolic regulator, secreted during inflammation, may take part in the pathogenesis of IBD (Liu et al., 2017; Liu et al., 2023). It has been shown that dextran sulfate sodium-induced colitis resulted in increased expression of FGF21, while the absence of FGF21 alleviated colitis symptoms, reduced adipose tissue lipolysis and prevented weight loss (Liu et al., 2017; Liu et al., 2023). Furthermore, also in IBD patients the acute phase of the disease was found to be associated with a significant increase in serum FGF21 level (Tomasik et al., 2010; Liu et al., 2017). While low body weight in IBD has multifactorial pathogenesis, it may be hypothesized that higher FGF21 level could contribute to the state of malnutrition. Colitis-induced FGF21 expression may subsequently activate lipolysis in WAT and weight loss (Liu et al., 2017). However, the pathophysiological link between IBD and FGF21 remains to be unraveled.

The aim of the current study was to assess the correlation between serum FGF21 level and inflammatory markers such as CRP and fecal calprotectin as well as indicators of nutritional status in patients with IBD.

## 2 Materials and methods

### 2.1 Study design

This cross-sectional study was performed at the Department of Gastroenterology and Hepatology of Wroclaw Medical University (Poland) between January 2021 and March 2023. All enrolled patients underwent a detailed clinical interview to assess their symptoms, associated disorders, and current treatment. The patients underwent also routine diagnostic assessments, including a physical examination and fasting blood laboratory tests. Additionally, they provided stool samples to determine calprotectin content. In patients with clinical indications, colonoscopy and/or enterography were also carried out.

Clinical and endoscopic activity of IBD was evaluated based on validated scales and indices. The Rachmilewitz index (Rachmilewitz, 1989) and the Mayo Endoscopic Score (Rubin et al., 2019) were applied to UC patients. The Crohn’s Disease Activity Index (CDAI) (Freeman, 2008) and the Simple Endoscopic Score for CD (SES-CD) (Daperno et al., 2004) were used in CD patients. Patients were categorized as being in either active or inactive phase of the disease based on the assessment of clinical, laboratory, and endoscopic criteria. Patients who fulfilled the criteria outlined as fecal calprotectin level lower than 200 μg/g, 0–4 points in the Rachmilewitz index and 0–2 points in the Mayo Endoscopic Score (for UC patients), the CDAI score lower than 200 points, and the SES-CD score lower than 7 points (for CD patients) were categorized as being in remission. All other subjects were assigned to the active phase group. CD patients with active changes in enterography were automatically included in the active phase group.

### 2.2 Subjects

Among 105 IBD patients there were 31 patients with active UC, 16 patients with inactive UC, 36 patients with active CD, and 22 patients with inactive CD. The control group consisted of 17 healthy volunteers without gastroenterological symptoms. To exclude undiagnosed IBD or other intestinal inflammation, fecal calprotectin test was performed in all controls.

The exclusion criteria were as follows: pancreatitis, chronic liver diseases (except single cysts and steatosis), diabetes, body mass index (BMI) ≥ 30 kg/m^2^, treated hyperlipidemia, ischemic heart disease, chronic kidney diseases (except single cysts and kidney stones), malignancies, alcohol dependence syndrome, history of abdominal surgical procedures (except appendectomy and procedures related to IBD).

### 2.3 Quantitative evaluation of serum FGF21 and fecal calprotectin levels

The fasting blood and stool samples provided by participants were stored at −80°C until the analysis. The quantitative evaluation of serum FGF21 [pg/ml] and fecal calprotectin [μg/g] levels were performed by immunoenzymatic methods: Human FGF-21 ELISA (BioVendor, Laboratorni medicina a.s., Czech Republic) and EK-CAL (Bühlmann Laboratories, Switzerland), respectively.

### 2.4 Statistical analysis

The obtained individual results are presented as numbers, percentage, mean values with standard deviation (±SD), or medians with the lower and upper quartiles (Q1-Q3). The normality of data was determined using the Shapiro-Wilk test. To compare quantitative variables with a normal distribution we assessed the homogeneity of variances using the Levene test, and then conducted a Student’s t-test (no significant variance difference) or a *t*-test with independent variances (significant variance heterogeneity), respectively. The Mann-Whitney U test compared quantitative variables with abnormal distribution. To compare categorical variables, the assumption of expected frequencies was checked – values less than 5 in a maximum of 20% of cell fields for the chi-square test, and proceed with Pearson’s chi-square test of independence or the Fisher exact test, respectively. The Spearman rank correlation coefficient and Kendall Tau correlations were calculated to test associations between variables. The statistical significance level was set at *p* < 0.05.

## 3 Results

### 3.1 Group characteristics

The characteristics of the studied groups of patients are presented in Table 1. The whole group of IBD patients included 68 males (65%) and 37 females (35%), at median age of 33 (27–41) years. There were no significant differences between the patient groups with respect to gender and age. The median duration of the disease was 63 (13–132) months. Of note, subjects with active UC were characterized by the shortest median disease duration amounting to 18 months and 39% of those patients were diagnosed with IBD within 1 year preceding the study. The control group (*n* = 17) included 9 males and 8 females, at median age of 28 (27–30) years and mean BMI of 22.4 ± 2.8 kg/m^2^.

**TABLE 1:** Detailed characteristics of the studied patient groups.

	Active UC (Group 1)	Inactive UC (Group 2)	Active CD (Group 3)	Inactive CD (Group 4)	*p* 1 vs. 2	*p* 3 vs. 4
Group characteristics						
n	31	16	36	22		
Men, n (%)	23 (74.2)	9 (56.3)	23 (63.9)	13 (59.1)	0.211	0.715
Age, median (Q1-Q3)	36 (26–40)	33.5 (24–45.5)	31 (27–40.5)	33 (28–45)	0.955	0.386
Duration of the disease (months), median (Q1-Q3)	18 (1.5–60)	58 (13–108)	90 (20.5–162)	120 (84–192)	0.099	0.161
BMI, mean ± SD, kg/m^2^	21.8 ± 3.6	22.2 ± 4.2	21.9 ± 4	22.9 ± 2.9	0.704	0.285
IBD activity						
Rachmilewitz index median (Q1-Q3)	9 (5–12)	1.5 (0–4)	–	–	<0.001	–
Mayo Endoscopic Score median (Q1-Q3)	3 (2–3)	0 (0–1)	–	–	<0.001	–
CDAI median (Q1-Q3)	–	–	266.6 (179.3–385.5)	77.9 (33.5–113.9)	–	<0.001
SES-CD mean ± SD	–	–	7.7 ± 4.8	4.2 ± 3.0	–	0.008
Treatment						
Mesalamine, n (%)	31 (100)	15 (93.8)	23 (63.9)	12 (54.5)	0.340	0.480
Steroids, n (%)	23 (77.4)	3 (18.8)	17 (47.2)	3 (13.6)	<0.001	0.008
Azathioprine, n (%)	9 (29.0)	3 (18.8)	13 (36.1)	5 (22.7)	0.505	0.285
Biological treatment, n (%)	1 (3.2)	2 (12.5)	2 (5.6)	3 (13.6)	0.264	0.357
Antibiotics, n (%)	8 (25.8)	3 (18.8)	12 (36.3)	1 (4.5)	0.725	0.009
Probiotics, n (%)	6 (19.4)	2 (12.5)	7 (19.4)	4 (18.2)	0.697	1.000

UC, ulcerative colitis; CD, Crohn’s disease, BMI, body mass index; CDAI, Crohn’s Disease Activity Index, SES-CD, Simple Endoscopic Score for Crohn’s disease.

Variables are presented as number (n) with percentage (%), mean values with standard deviation (±SD), or medians with the lower and upper quartiles (Q1-Q3).

The results of the evaluation of clinical and endoscopic IBD activity based on the applied scales and indices are presented in Table 1.

In most cases, the patients in an active phase of IBD were administered steroids. Three patients with inactive UC and 3 with inactive CD were still on steroids while tapering their dose. They were admitted to the hospital for check-ups after an exacerbation of the disease which had occurred 2–3 months earlier.

The results of blood and stool tests in IBD patients including the evaluation of serum CRP and fecal calprotectin are presented in Table 2. Mean fecal calprotectin in the control group amounted to 15.3 ± 10.3 μg/g.

**TABLE 2:** Laboratory test results in the groups of studied patients.

	Active UC (Group 1)	Inactive UC (Group 2)	Active CD (Group 3)	Inactive CD (Group 4)	*p* 1 vs. 2	*p* 3 vs. 4
Fecal calprotectin [µg/mL], mean ± SD	1528.7 ± 673.5	78.9 ± 61.2	1266.7 ± 733.9	74.9 ± 41.9	<0.001	<0.001
CRP [mg/L], median (Q1-Q3)	16.6 (5.2–46.8)	1.8 (0.9–6.9)	7.9 (4.3–24.6)	2.1 (1.2–7.0)	<0.001	0.001
Total cholesterol [mg/dL], mean ± SD	143.5 ± 39.3	194.1 ± 34.2	148.1 ± 37.3	160.9 ± 54.9	<0.001	0.046
LDL [mg/dL], mean ± SD	85.1 ± 27.7	115.4 ± 36.3	79.1 ± 30.6	97.5 ± 35.9	0.002	0.042
HDL [mg/dL], mean ± SD	41.3 ± 13.9	53.3 ± 15.0	49.2 ± 13.7	55.0 ± 15.4	0.009	0.142
Triglycerides [mg/dL], median (Q1-Q3)	104.5 (67–125)	88.5 (65–122)	91.0 (69–108)	102.5 (72–122)	0.308	0.380
Total protein [g/dL], mean ± SD	6.1 ± 0.9	7.2 ± 0.7	6.7 ± 0.7	7.12 ± 0.4	<0.001	0.022
Albumin [mg/dL], mean ± SD	3.5 ± 0.6	4.3 ± 0.5	3.8 ± 0.5	4.2 ± 0.4	<0.001	0.003
Hemoglobin [g/dL], mean ± SD	11.6 ± 1.6	14.1 ± 1.9	12.3 ± 2.3	13.6 ± 2.1	<0.001	0.035
Iron [μg/dL], median (Q1-Q3)	31 (17–50)	85 (68–114)	36 (17–84)	70 (25–96)	<0.001	0.194
Ferritin [µg/L], median (Q1-Q3)	54.6 (10.6–249.8)	90.9 (51.2–182.5)	46.8 (20.9–158.8)	23.7 (15.5–67.9)	0.541	0.067
Transferrin [g/L], median (Q1-Q3)	2.4 (1.6–2.7)	2.4 (2.2–2.7)	2.3 (2.0–2.8)	2.6 (2.5–2.9)	0.189	0.011
Vitamin D [ng/mL], median (Q1-Q3)	20.7 (10.0–26.2)	27.9 (17.3–42.0)	24.8 (16.9–33.3)	29.3 (15.5–33.5)	0.103	0.684

UC, ulcerative colitis; CD, Crohn’s disease, LDL, low-density lipoprotein; HDL, high density lipoprotein.

Variables are presented as mean values with standard deviation (SD), or medians with the lower and upper quartiles (Q1-Q3).

### 3.2 Serum FGF21 level

**FIGURE 1:**
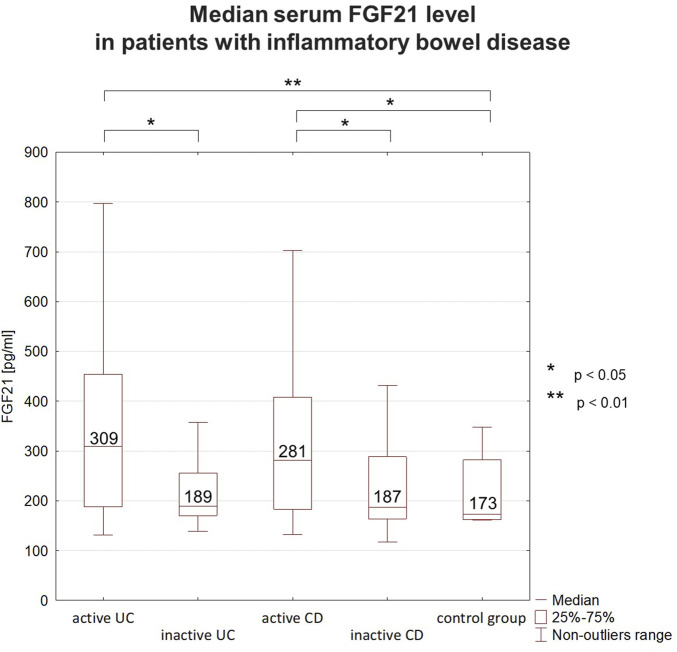
Median serum FGF21 levels in patients with inflammatory bowel disease (IBD). In IBD patients FGF21 level fluctuated depending on the disease activity. Higher concentrations were observed during flares than during remission. FGF21 concentrations during remission were comparable to those in the control group. FGF21 - fibroblast growth factor 21, UC–ulcerative colitis, CD–Crohn’s disease.

Analyzing the serum FGF21 level in IBD patients, a clear tendency for higher values in active IBD phase was observed. The median serum FGF21 level was higher in active UC than in inactive UC, as well as in the control group. However, there was no significant difference in FGF21 level between patients with inactive UC and the controls. The same fluctuation depending on disease activity was observed in the group of CD patients (Figure 1). An elevated concentration of FGF21 (>300 pg/mL) was detected in 39 patients that accounted for 37% of all IBD patients. Most of those subjects (32/39, 82%) were in the active phase. The increased FGF21 level was found in 48% of patients with active UC and 47% of patients with active CD. FGF21 values below the normal range were not detected in any of the studied IBD patients.

### 3.3 Nutrient deficiencies in IBD patients

Evaluating the nutritional status, underweight (BMI <18.5 kg/m^2^) was diagnosed in 17% of the IBD patients. Weight loss exceeding 5% of body mass within 1 month was documented in 21% of the subjects. Selected nutrient deficiencies detected in the studied patients are presented in Table 3. Almost 30% of subjects with active UC and active CD had low cholesterol level. Almost 40% of patients with active UC and 17% of patients with active CD had hypoproteinemia. More than 50% of patients with active UC and almost 30% of patients with active CD had hypoalbuminemia. Iron deficiency and anemia was also commonly observed. Hemoglobin levels lower than 11.9 g/dL in women and lower than 12.9 g/dL in men were reported in 81% of patients with active UC, 25% with inactive UC, 44% with active CD, and 32% with inactive CD. Vitamin D deficiency was confirmed in 64% of the patients, with a deep deficiency (<10 ng/mL) identified in 14% of them. Nutritional deficiencies were more often observed in patients with active UC than those with inactive UC, except for low vitamin D levels. In contrast, these deficiencies occurred at similar rates for patients with CD regardless of whether the disease was active or inactive, except for low albumin levels, which were more prevalent in active CD.

**TABLE 3:** Nutrient deficiencies in the studied IBD patients.

	Active UC (Group 1)	Inactive UC (Group 2)	Active CD (Group 3)	Inactive CD (Group 4)	*p* 1 vs. 2	*p* 3 vs. 4
n	31	16	36	22	-	-
Hypocholesterolemia, n (%)	9 (29.0)	0 (0.0)	10 (27.8)	3 (13.6)	0.019	0.332
Hypoproteinemia, n (%)	12 (38.7)	1 (6.3)	6 (16.7)	0 (0.0)	0.036	0.073
Hypoalbuminemia, n (%)	16 (51.6)	1 (6.3)	10 (27.8)	1 (4.6)	0.003	0.039
Iron deficiency, n (%)	19 (61.3)	3 (18.8)	23 (63.9)	12 (54.6)	0.007	0.480
Vitamin D deficiency, n (%)	24 (77.4)	8 (50.0)	24 (66.7)	11 (50.0)	0.056	0.208

Hypocholesterolemia (total cholesterol level <125 mg/dL), hypoproteinemia (total protein level <6 g/dL), hypoalbuminemia (albumin level <3.5 mg/dL), iron deficiency (ferritin level <30 μg/L in inactive phases and <100 μg/L in active phases), and vitamin D deficiency (25(OH)2D3 level <30 ng/mL).

UC, ulcerative colitis; CD, Crohn’s disease.

### 3.4 The analysis of correlation between serum FGF21 level and studied variables

There was no significant correlation of serum FGF21 level with the clinical and endoscopic disease activity indices (Table 4).

**TABLE 4:** The analysis of correlations between serum FGF21 level and the studied variables.

Variable	UC		CD	
	Correlation coefficient	*p*	Correlation coefficient	*p*
Rachmilewitz index	0.19	0.203	–	–
Mayo Endoscopic Score^*^	0.20	0.080	–	–
CDAI	–	–	0.25	0.057
SES-CD	–	–	0.20	0.216
Fecal calprotectin	0.53	0.001	0.29	0.047
CRP	0.21	0.165	0.27	0.036
Total cholesterol	−0.33	0.028	−0.27	0.035
Triglycerides	0.38	0.009	0.07	0.615
Total protein	−0.43	0.003	−0.33	0.011
Albumin	−0.40	0.006	−0.37	0.004
Iron	−0.14	0.337	−0.39	0.002
Hemoglobin	−0.37	0.010	−0.21	0.113
Vitamin D	−0.21	0.175	−0.32	0.014
BMI	−0.29	0.047	−0.22	0.091

UC, ulcerative colitis; CD, Crohn’s disease, CDAI, Crohn’s Disease Activity Index, SES-CD, Simple Endoscopic Score for Crohn’s disease, BMI, body mass index.

The Spearman rank correlation coefficient and Kendall Tau Correlations (^*^) were calculated to test associations between variables.

A significant positive correlation between FGF21 and fecal calprotectin levels were identified in both UC and CD, along with a correlation between FGF21 and CRP levels in CD patients, but not in UC patients (Table 4).

Negative correlations were observed between FGF21 level and total cholesterol, protein, and albumin levels in both UC and CD patients. Moreover, in UC patients, negative correlations of FGF21 level with hemoglobin level and BMI were noticed, and a positive correlation between FGF21 and triglyceride levels. In CD patients negative correlations of FGF21 level with iron and vitamin D levels were detected (Table 4).

## 4 Discussion

In this study, we have confirmed that serum FGF21 levels are higher in patients with active IBD, both UC and CD, compared to patients with inactive IBD and healthy controls. The available data on FGF21 level fluctuations in adult IBD patients are scarce. In one study higher plasma FGF21 levels in IBD patients compared to healthy controls were reported; however, the investigated group of patients was not divided into subjects with active and inactive disease (Liu et al., 2017). In another study conducted in children with IBD, serum FGF21 level was higher in the disease flare with a subsequent significant decrease after treatment (Tomasik et al., 2010). Additional compelling evidence regarding the role of FGF21 in intestinal inflammation comes from studies in animal models which show that chemically induced colitis is one of the factors stimulating the secretion of FGF21 (Liu et al., 2017; Liu et al., 2023; Al-Aqil et al., 2018). In an experimental IBD model, Liu et al. (2023) demonstrated that endogenous FGF21 was increased in dextran sulfate sodium-induced colitis, which contributed to the progression of the disease and a significant loss of body weight. The abovementioned study has also shed light on one of the potential mechanisms contributing to the FGF21 action. In FGF21 knockout mice, FGF21 depletion attenuated the severity of chemically induced acute colitis by enhancing the activation of the interleukin 22-STAT3 signaling pathway in intestinal epithelial cells (Liu et al., 2023).

The identification of FGF receptors and β-Klotho co-receptors within intestinal tissues supports the hypothesis that FGF21 plays a role in intestinal pathophysiology of IBD (Danopoulos et al., 2017; Aaldijk et al., 2023). Indeed, an immunohistochemistry analysis revealed that in the intestines of UC patients there is increased FGF21 expression located in the extra-epithelial compartment, while increased β-Klotho co-receptor expression is observed mainly on the surface of the intestinal epithelium (Muise et al., 2008; Rydén, 2009).

The current results not only confirm previous observations, but further indicate that FGF21 level correlates with the intensity of intestinal inflammation reflected by fecal calprotectin level. Moreover, the association between FGF21 and CRP levels is also present in CD patients. The fact that no correlation was found between FGF21 and CRP levels in UC patients may be related to quite wide range of CRP values in that group, particularly in active phase of disease. Additionally, it has been suggested that in UC, fecal calprotectin level has a better significance for detecting colitis compared to CRP (Anindita et al., 2023). Noteworthy, other researchers (Gariani et al., 2013) demonstrated a correlation between FGF21 level and CRP level in patients with systemic inflammatory response syndrome and had even proposed FGF21 as a non-specific marker for systemic inflammation.

A novel aspect of the current research is related to the evaluation of potential correlation between serum FGF21 level and validated clinical and endoscopic disease activity scales and indices in IBD. However, despite the observed fluctuations in FGF21 level in active *versus* inactive IBD phases, as well as the presence of correlation between FGF21 and inflammatory markers, no significant association of FGF21 level with validated disease activity scales and indices were found. Nevertheless, FGF21 as an inflammatory marker doesn’t have to correlate directly with more complex scales and indices of clinical or endoscopic activity of the diseases.

Given the role of FGF21 as an endocrine metabolic regulator that is expressed in many metabolically active tissues such as the liver and WAT as well as its concomitant involvement in intestinal inflammation, one of the aims of the current study was to assess the relation between FGF21 level and nutritional status parameters in IBD patients. Among the studied IBD patients, 17% of subjects were underweight. A significant percentage of patients were characterized by hypocholesterolemia, hypoproteinemia, hypoalbuminemia, iron deficiency anemia and vitamin D deficiency. The observed deficiencies in our study were more prevalent compared to some previous reports (Casanova et al., 2017; Prieto et al., 2021). Particularly frequently nutritional disturbances were detected in patients with active UC. To some extend it could be related to the relatively short duration of the disease (many patients in that group were only recently diagnosed). In fact, according to the available data, malnutrition is more prevalent among patients with a recently diagnosed IBD (Gold et al., 2023). Furthermore, patients with UC are more prone to develop nutritional deficiencies during active phase of the disease, whereas subjects with CD typically develop features of malnutrition gradually over an extended period of time (Balestrieri et al., 2020). Regarding a high rate of vitamin D deficiency in the studied population (amounting to 64% of the subjects), it was comparable to the results of previous studies performed in IBD patients in Poland (Krela-Kaźmierczak et al., 2015; Tulewicz-Marti et al., 2022). Importantly, vitamin D3 deficiency affected a large percentage of IBD patients in remission.

While analyzing the associations between FGF21 level and selected nutritional status parameters, we found that FGF21 level negatively correlated with BMI, total cholesterol, total protein, albumin, iron, hemoglobin and vitamin D levels. A positive correlation of FGF21 and triglyceride levels may be associated with increased lipolysis activity.

The spectrum of nutritional disturbances in IBD patients is wide. On one hand, malnutrition is a major complication of IBD and it is primarily responsible for chronic weight loss (Scaldaferri et al., 2017). On the other hand, numerous recent studies have linked IBD to metabolic syndrome, which includes diabetes, obesity, and dyslipidemia, as they share some common pathophysiological links including inflammation, adipose tissue dysregulation, and gut dysbiosis (Michalak et al., 2016; Szilagyi, 2020; Verdugo-Meza et al., 2020). Experimental and clinical evidence supports parallels between metabolic nature of gut inflammation in IBD and the inflammatory state in metabolic diseases (Adolph et al., 2022). Specifically, the role of adipose tissue in the development of metabolic syndrome and IBD has been extensively studied (Choe et al., 2016). Noteworthy, in various metabolic disorders such as obesity, hyperlipidemia, diabetes, and metabolic dysfunction-associated steatotic liver disease, elevated serum FGF21 level were also recorded (Yang et al., 2023). However, in these conditions the FGF21 resistance seems to occur (Aaldijk et al., 2023).

A recently published meta-analysis of clinical trials demonstrated that treatment with FGF21 analogues significantly reduces total cholesterol level and contributes to weight loss (Carbonetti et al., 2023). Additionally, several other studies propose FGF21 as potential therapeutic agent with anti-inflammatory effect (Feingold et al., 2012; Singhal et al., 2016; Wang et al., 2018). Contrary, an increase in FGF21 level during the active phase of IBD exerts negative metabolic effects contributing to malnutrition and weight loss and exacerbating inflammation. This discrepancy may be explained by the possible dual action of FGF21. In fact, FGF21 has the capability to either suppress or promote lipolysis in WAT, depending on whether the person is during fasting or in the postprandial state, respectively (Xie et al., 2020). Similarly, pro- or anti-inflammatory action of FGF21 may depend on the given pathophysiological state such as acute *versus* chronic inflammation. In experimental studies, it has been shown that overexpression of FGF21 may reduce hepatic cholesterol production (Huang et al., 2017), increase the liver production of bile salts from cholesterol (Al-Aqil et al., 2018), induce the lipolysis of adipose tissue (Liu et al., 2017), reduce muscle mass and strength (Oost et al., 2019). Therefore, modulation of FGF21 signaling pathway could emerge as a target in IBD and related metabolic disorders. However, the effects of exogenous FGF21 treatment on acute and chronic colitis and colitis recovery have not been adequately examined so far.

Among limitations of the study is its cross-sectional character. Furthermore, the effects of used medications may constitute confounding factors affecting the results. For example, steroid therapy leads to enhanced FGF21 expression in the liver (Vispute et al., 2017; Al-Aqil et al., 2018). However, since many studies have confirmed that inflammatory stimuli are inducers of FGF21 expression, the use of steroids most likely only contributes to the FGF21 level increase, but is not solely responsible for the effect. Additionally, to further explore the association between FGF21 and nutritional status, it would be useful to perform more detailed analysis including the evaluation of fat mass index, fat-free mass index or muscle strength. It might be important given that some IBD patients with sarcopenia have normal BMI values (Scaldaferri et al., 2017; Balestrieri et al., 2020). The novelty of the study is related to the pioneer report on FGF21 level fluctuations in adult IBD patients in active and inactive phases of the disease as well as on the correlation of FGF21 level with inflammatory markers and nutritional status parameters.

In conclusion, our results show that FGF21 level correlates directly with the intensity of intestinal inflammation and inversely with nutritional status of IBD patients. Therefore, the multifactorial pathogenesis of malnutrition and weight loss in IBD patients may be related to disturbances in FGF21 level. Further studies are warranted to clarify the exact mechanism of complex action of FGF21 within the gut-liver axis to unravel potential new therapeutic targets in IBD and related metabolic disturbances.

## Data Availability

The raw data supporting the conclusion of this article will be made available by the authors, without undue reservation.
